# Guardians of the Game: UV‐Specific Skin Cancer Prevention by Coaches in Outdoor Sports

**DOI:** 10.1111/phpp.70007

**Published:** 2025-01-20

**Authors:** Sophie Leer, Clara Ehls, Sven Schneider

**Affiliations:** ^1^ Center for Preventive Medicine and Digital Health, CPD, Division of Public Health, Social and Preventive Medicine, Medical Faculty Mannheim Heidelberg University Heidelberg Germany

**Keywords:** coaches, health risks, outdoor sports, physical activity, skin cancer

## Abstract

**Background:**

Due to the global rise in UV radiation, the prevalence of skin cancer is increasing significantly, with outdoor athletes being identified as a particularly vulnerable population group.

**Methods:**

This nationwide, cross‐sectional study was conducted among adult coaches from the 10 largest outdoor sports associations in Germany. Their applied prevention measures and the potential for further improvement in prevention were evaluated by guideline based scores (range [0–100]). Additionally, sport‐, coach‐, and club‐specific variables were analysed and barriers that prevent comprehensive UV prevention were identified.

**Results:**

The UV prevention practice of the 1200 participating coaches averaged 53.43 ± 16.37 [0.00–95.31], while the potential UV prevention options were assessed at a comparatively higher value of 58.82 ± 17.53 [0–100]. Notably, the proper use of sunscreen emerged as the most neglected preventive measure. Coaches identified the greatest potential for improvement in sunscreen renewal, water‐resistant sun protection products and sunglasses. The study also revealed significant differences in UV protection practices between different sports, with soccer, tennis, and swimming exhibiting the most pronounced deficits. Factors such as coach qualification and experience, as well as club size and the size of training groups influenced the level of UV protection implemented. Many coaches cited various implementation obstacles such as fixed training times and resource constraints.

**Conclusion:**

The study highlights specific areas for improvement in UV protection practices in outdoor sports, considering differences on sport, coach, and club levels. The significant number of active athletes in these sports underscores the public health importance of addressing UV protection in this field.

## 1 Introduction

According to the World Health Organization and current medical guidelines, outdoor athletes are among the main risk groups for skin cancer [1, 2, 3]. League matches, middle‐distance competitions, athletics competitions, tennis, riding and golf tournaments, regattas and skiing competitions cannot simply be moved into the shade or indoors. Training and competitions often take place even during the strongest sunlight at midday or in environments with particularly high UV exposure (e.g., with reflection from sand, water, or snow) [4, 5]. Furthermore, competition guidelines in many areas of sports prevent the use of protective clothing such as headgear, sunglasses, or long‐sleeved clothing [5]. And finally, sports‐related sweat production increases the skin's photosensitivity [5, 6].

This specific risk constellation can lead to higher incidences of UV‐related skin cancer: According to a longitudinal study, young outdoor athletes had a significantly higher increase of melanocytic nevi over the course of 2 years compared to children of the same age who did not exercise outdoors [7].

In principle, UV‐related illnesses are completely avoidable through adequate prevention. The most important key people in this context are the coaches. Due to the long latency period for the development of skin cancer, it is of the utmost importance for them to provide early and consistent protection against serious UV risks for the athletes in their care, who are often minors [3, 4, 5, 8].

For this reason, we conducted a nationally representative study asking outdoor sports coaches to what extent the preventive measures recommended in the relevant guidelines for protection against solar UV radiation are implemented in their respective training practices. We were particularly interested in discovering whether prevention practices vary between sports and other coach‐ and club‐specific factors and which barriers prevent comprehensive UV prevention.

## 2 Methods

### 2.1 Study Design and Setting

The data basis for this study was a nationwide representative cross‐sectional survey of outdoor sports coaches: the “C^3^O study” (Climate Change, Coaches and Outdoor Sports–Study). We utilized the definition of “outdoor sports” presented by Dee et al. [9], which states that a sport is an outdoor sport if the competitions are typically held outdoors. According to this definition, the 10 largest outdoor sports associations in Germany can be found in the official membership statistics of the umbrella organisation, the German Olympic Sports Confederation [Deutscher Olympischer Sportbund] (DOSB) [10]: At the end of 2022, these were the German Football Association [Deutscher Fußball‐Bund] (DFB), the German Tennis Association [Deutscher Tennis Bund] (DTB), the German Alpine Association [Deutscher Alpenverein] (DAV), the German Athletes Association [Deutscher Leichtathletik‐Verband] (DLV), the German Equestrian Federation [Deutsche Reiterliche Vereinigung] (FN), the German Golf Association [Deutscher Golf Verband] (DGV), the German Life Saving Association [Deutsche Lebens‐Rettungs‐Gesellschaft] (DLRG), the German Ski Association [Deutscher Skiverband] (DSV), the German Sailing Association [Deutscher Segler‐Verband] (DSV), as well as the German Cyclists Federation [Bund Deutscher Radfahrer] (BDR). These sports associations had a total of 14 million members on the above‐mentioned reporting date, which corresponded to 16% of the German population.

In order to reach as many coaches as possible, the cross‐sport and sport‐specific federal and state associations were contacted and the call for participation in the study was distributed via the websites of the associations, the printed association notices, and the e‐mail and telephone directories of the sports associations. The survey was conducted as an asynchronous computer aided web interview (CAWI) with the help of a standardized questionnaire using the Lime‐Survey software package during the time span of May 2022–June 2023. Before the start of the survey phase, the online questionnaire was subjected to an expert review (*n* = 4) and a classic pretest (*n* = 8). An individualized web link contained information on the content and procedure of the study, the informed consent form as well as access to the final online questionnaire. The study follows the Declaration of Helsinki and this publication was conducted in accordance with the STROBE statement [11]. Prior to the start of the study, the responsible ethics committee at Heidelberg University gave a positive vote for the approval of the study (AZ 2021‐653, November 16, 2021). This process was followed by a pre‐registration in the German Clinical Trials Registry [Deutsches Register Klinischer Studien] (DRKS; Registration number DRKS00027815; https://drks.de/search/de/trial/DRKS00027815 from January 18, 2022).

### 2.2 Participants

Inclusion criteria were coaching in competitive or popular sport in a German club in one of the 10 major outdoor sports clubs listed above, being of legal age, and understanding German. The study participants were recruited by means of quota sampling, a systematic, non‐probabilistic sampling method, according to sport and federal state. The recruitment strategy described above resulted in a response of *n* = 1200 participants, which far exceeded the minimum sample size of *n* = 600 originally envisaged in the study protocol. Using a standard weighting procedure (so‐called “standard redressement”) [12], each of the 10 outdoor sports was represented with 120 cases (disproportionate quotas by sport) and the sample was representative nationwide with regard to the distribution across the 16 German federal states (proportional quotas by federal state).

### 2.3 Variables and Measurement

#### 2.3.1 Prevention Practice

**FIGURE 1:**
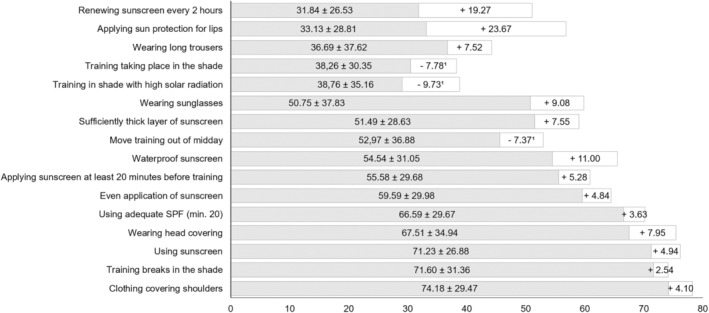
Implemented and possible UV protection measures in German outdoor sports. *N* = 1200. Light grey = total score “UV prevention practice.” White = potential for improvement with regard to UV prevention options. *Note*: 1) The actual implementation is better than expected for the trainer interviewed.

The recording of preventive measures to protect against UV radiation during training was based on the current S3 guideline for the prevention of skin cancer in Germany [2]. This guideline was published in September 2021 by the Association of the Scientific Medical Societies in Germany [Arbeitsgemeinschaft der Wissenschaftlichen Medizinischen Fachgesellschaften e. V.] (AWMF), the German Cancer Society [Deutsche Krebsgesellschaft e. V.] (DKG) and the German Cancer Aid [Deutsche Krebshilfe] (DKH). A further 44 medical institutions and specialist associations were involved. In Germany, an S3 guideline represents the highest quality level for a medical guideline. To operationalise our research question, we included 14 of the measures recommended in this guideline for protection against solar UV radiation in our questionnaire (guideline recommendations 5.2–7.6; see Figure 1 for details). As a stimulus, the coaches were given a vignette in the questionnaire, namely a “cloudless, sunny summer day.” Against this backdrop, they were asked to indicate on a 100‐point Likert scale with the poles 0 = “never” versus 100 = “always” how often each measure is currently implemented in a typical training session under these conditions. In order to take the specific situation of skiing into account, a “cloudless sunny day” was specified as a vignette for high UV exposure. The information on the individual prevention measures was then combined into an unweighted interval‐scaled sum score “UV prevention practice” with a range of [0–100].

#### 2.3.2 UV Prevention Options

The question was then asked: “Please think again about the typical training situation mentioned at the beginning, that is, a cloudless, sunny (summer) day. How easy is/would it be to implement the following statements in the training you lead?” The 14 measures in question were presented again with the same wording. Analogous to the procedure above, a sum score “UV prevention options” was calculated.

In addition, three further measures recommended in the guidelines for protection from solar UV radiation were asked by means of nominal questions: Here, the coaches were asked to provide information on whether they inform the athletes about the UV index during training on a cloudless, sunny day (in accordance with guideline recommendation 7.3), whether the club provides free protection from UV radiation on such days, such as sunscreen, headgear, or sunglasses (in accordance with guideline recommendation 7.4) and whether the club takes sun protection into account when planning events, for example, by scheduling them outside the midday hours, providing artificial shading, etc. (in accordance with guideline recommendation 7.5).

#### 2.3.3 Barriers

Finally, coaches were able to provide detailed free text information on why they felt “certain prevention measures are very difficult to implement”.

### 2.4 Independent Variables (Determinants)

In addition to the type of sport, coach‐specific (gender, age, qualification, experience, and performance level) and club‐specific (age and size of the training group, size of the club, frequency of training and geographical region of the club) factors were considered as possible determinants of our explananda.

### 2.5 Statistical Analysis

The C^3^O study was analysed using classic descriptive (incl. arithmetic mean, standard deviation, median, range) and inferential methods with the aid of the IBM SPSS Statistics 29.0.0 programme (IBM Corp., Armonk, USA). The interval‐scaled scores were approximately normally distributed. The differences between the arithmetic mean and median were well below 0.1 standard deviations in each case. All tests were exploratory and two‐sided with a significance threshold of *p* ≤ 0.05. Group differences were analysed in a first step using *t*‐tests and ANOVA, bivariate significant determinants in a second step using multiple linear regression analyses.

The method of structuring, qualitative content analysis according to Mayring was used to analyse the self‐reported barriers [13]. For this purpose, CE pre‐coded the first 120 statements of the interviewees (approximately 10% of the total material) in order to derive higher‐level categories inductively. This initial coding was followed by the creation of a coding guide including anchor examples and coding rules. The material was then read through twice, with all respondents' statements assigned to the categories. The coding guide, including anchor examples and coding rules, is available from the corresponding author.

### 2.6 Bias and Quality Control

The online survey method made it possible to carry out plausibility checks during the entry process, for example by not accepting unauthorised values and incompletely completed questionnaires. Multiple entries from the same IP address were also not permitted. In addition, plausibility checks were carried out in the data set after the data base lock on June 30, 2023. This did not reveal any systematic anomalies. The average processing time was 24 min and over 95% of respondents took between 10 and 60 min to complete the questionnaire.

In addition, the qualitative content analysis was checked for intercoder reliability. For this purpose, the responses of a random selection of 200 interviewees were coded blinded by a second trained rater (SL). This resulted in a Cohen's Kappa of 0.694 (*p* < 0.001), which indicates “substantial” [14] or “good” [15] reliability.

## 3 Results

The 1200 coaches surveyed across Germany were on average 44 (±14, range [18–81]) years old and 32% female. The participants could look back on an average of 15 years' experience as coaches. Their training groups comprised an average of 11 (±8) people. A quarter (26%) led training for children, 40% for adolescents, and 34% for adults.

The total score, which summarises UV prevention practice, was on average 53.43 (±16.37, range [0.00–95.31]). The basic UV prevention options were rated with a comparatively higher score of 58.82 (±17.53, range [0.00–100.00]). The proper use of sunscreen was treated with the most negligence: repeated application of sunscreen (31.84) and the use of UV protection for the sensitive lip area (33.13) are rarely common practice. Coaches also rarely move their training sessions to the shade, either in principle (38.26) or at least at lunchtime (38.76; Figure 1). Trainees are most likely to wear clothing that covers their shoulders during training (74.18) and to seek shade during breaks (71.60; Figure 1). From the coaches' point of view, the greatest potential for improvement lies in a better use of sunscreen, especially when reapplying cream (+19.27) and lip protection (+23.67). In terms of potential for improvement, the coaches also see room for improvement in the use of water‐resistant sun protection products (+11.00) and sunglasses (+9.08; Figure 1).

The coaches also report deficits regarding further protective measures in line with the guidelines: Only in 22.42% of cases is information about the current UV index provided during training on cloudless, sunny (summer) days. Even more rarely does the club provide free protection, for example in the form of sunscreen or similar (13.25%). At competitions and similar events, the club takes UV protection measures in 55.67% of cases.

**FIGURE 2:**
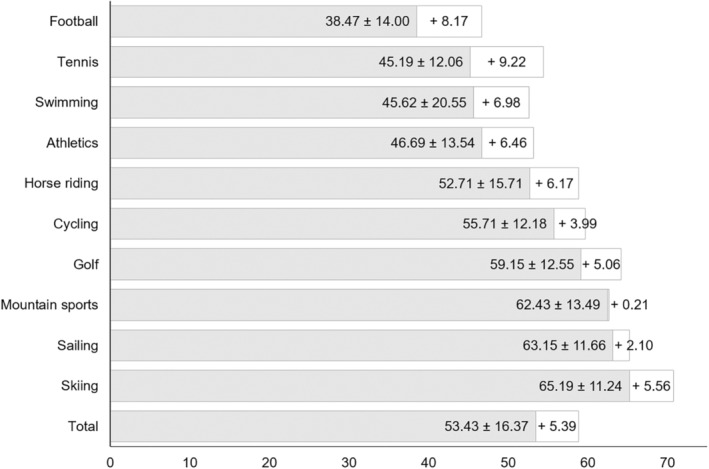
Prevention practice and potential for improvement in UV protection measures by type of sport. Legend: *n* = 1200. Light grey = total score “UV prevention practice.” White = potential for improvement with regard to UV prevention options.

The current prevention practice (ANOVA: *F*‐value: 51.43, *df* = 9, *p* < 0.001) and the possible options for action (ANOVA: *F*‐value: 23.70, *df* = 9, *p* < 0.001) differ significantly between the types of sport. The greatest deficits in the preventive measures applied are evident in football, tennis and swimming (Figure 2). When coaches are asked about unused opportunities for implementation, these sports also show the greatest potential for improvement (with values of +8.17, +9.22, and + 6.98; Figure 2). On the other hand, the potential seems to be more fully exploited in the sports with higher scores for current prevention practice, as can be seen from the comparatively smaller differences (Figure 2).

Finally, we investigated whether, in addition to the type of sport, coach‐ and club‐specific factors also determine prevention behaviour. Firstly, the regression coefficients in Model 1 confirm the differences between the individual sports known from the bivariate analysis (Table 1, Model 1). While the age and gender of the coach do not appear to determine training practice, the more qualified the coach, the more their protective behaviour gradually increases (Table 1, Model 2): The higher the licence level, the more protective the coaches are of their athletes. The same applies to experience: compared to novices, coaches with 5–9 years of training experience have significantly higher score values. The coefficients for the other categories of this variable have the same sign and are also significant in the final overall model. Furthermore, according to our data, UV protection is less pronounced in competitive sports than in popular sports (Table 1, Model 2). Club‐specific factors also determine the level of UV protection: there are significantly greater deficits in smaller clubs, in smaller training groups and in children's and youth training (Table 1, Model 3). Essentially, the same picture emerged in the final overall model, taking into account only previously significant variables (Table 1, Model 4).

**TABLE 1:** Multivariable linear regression analyses on the implementation of UV protection measures among German coaches according to sport‐, coach‐ and club‐specific variables.

Variable	Model 1 sport‐specific variables			Model 2 trainer‐specific variables			Model 3 club‐specific variables			Model 4 overall model		
	*b*‐coefficients	CI	*p*	*b*‐coefficients	CI	*p*	*b*‐coefficients	CI	*p*	*b*‐coefficients	CI	*p*
Skiing	Ref.											
Football	−26.72	[−30.25 to −23.19]	< 0.001							−26.42	[−30.35 to −22.49]	< 0.001
Tennis	−19.99	[−23.52 to −16.46]	< 0.001							−19.59	[−23.32 to −15.87]	< 0.001
Swimming	−19.56	[−23.09 to −16.03]	< 0.001							−20.22	[−23.97 to −16.48]	< 0.001
Athletics	−18.50	[−22.03 to −14.97]	< 0.001							−18.50	[−22.48 to −14.51]	< 0.001
Horse riding	−12.48	[−16.01 to −8.95]	< 0.001							−12.21	[−15.85 to −8.58]	< 0.001
Cycling	−9.48	[−13.01 to −5.95]	< 0.001							−9.34	[−13.04 to −5.64]	< 0.001
Golf	−6.03	[−9.57 to −2.50]	< 0.001							−5.52	[−9.28 to −1.78]	0.004
Mountain sports	−2.76	[−6.29 to +0.78]	0.126							−5.71	[−9.71 to −1.71]	0.005
Sailing	−2.03	[−5.57 to +1.50]	0.259							−2.00	[−5.71 to +1.71]	0.290
Women				Ref.								
Man				+0.62	[−1.41 to +2.64]	0.551						
Age: 18–24 years				Ref.								
Age: 25–34 years				−1.04	[−4.66 to +2.59]	0.575						
Age: 35–44 years				+0.02	[−3.69 to +3.73]	0.991						
Age: 45–54 years				+2.99	[−0.70 to +6.67]	0.112						
Age: ≥ 55 years				+1.64	[−2.10 to +5.38]	0.390						
Without license				Ref.								
C‐license				+3.30	[+0.56 to +6.04]	0.018				+2.15	[−0.22 to +4.51]	0.075
B‐license				+4.47	[+1.06 to +7.87]	0.010				+0.76	[−2.24 to +3.76]	0.620
A‐license/diploma				+7.28	[+3.23 to +11.34]	< 0.001				−0.22	[−3.91 to +3.47]	0.907
Experience as trainer: 0–4 years				Ref.								
Experience as trainer: 5–9 years				+3.22	[+0.17 to +6.27]	0.039				+2.66	[+0.02 to +5.30]	0.048
Experience as trainer: 10–19 years				+1.54	[−1.58 to +4.65]	0.334				+2.62	[+0.04 to +5.19]	0.046
Experience as trainer: 20–29 years				+1.01	[−2.44 to +4.47]	0.565				+3.45	[+0.82 to +6.09]	0.010
Trainer in amateur sports				Ref.								
Trainer in competitive sports				−4.63	[−6.86 to −2.41]	< 0.001				−1.35	[−3.45 to +0.76]	0.210
Target group: Children and teenagers							Ref.					
Target group: Adults							+3.99	[+2.04 to +5.93]	< 0.001	+1.01	[−0.84 to +2.87]	0.285
Group size: 1–14							Ref.					
Group size: 15–19							−8.13	[−10.86 to −5.40]	< 0.001	−0.03	[−2.78 to +2.72]	0.985
Group size: ≥ 20							−6.57	[−9.52 to −3.62]	< 0.001	+0.04	[−2.86 to +2.93]	0.979
Club size: < 2.500 members							Ref.					
Club size: ≥ 2.500 members							+5.36	[+2.62 to +8.10]	< 0.001	+3.78	[+1.01 to +6.56]	0.008
Non‐daily training							Ref.					
Daily training							−0.23	[−2.65 to +2.20]	0.855			
Northern Germany							Ref.					
Southern Germany							+1.36	[−0.52 to +3.24]	0.156			
Constant	65.19			48.71			52.71			61.41		
*R* ^2^	0.280			0.028			0.076			0.293		
*n*	1200			1195			1200			1200		

**FIGURE 3:**
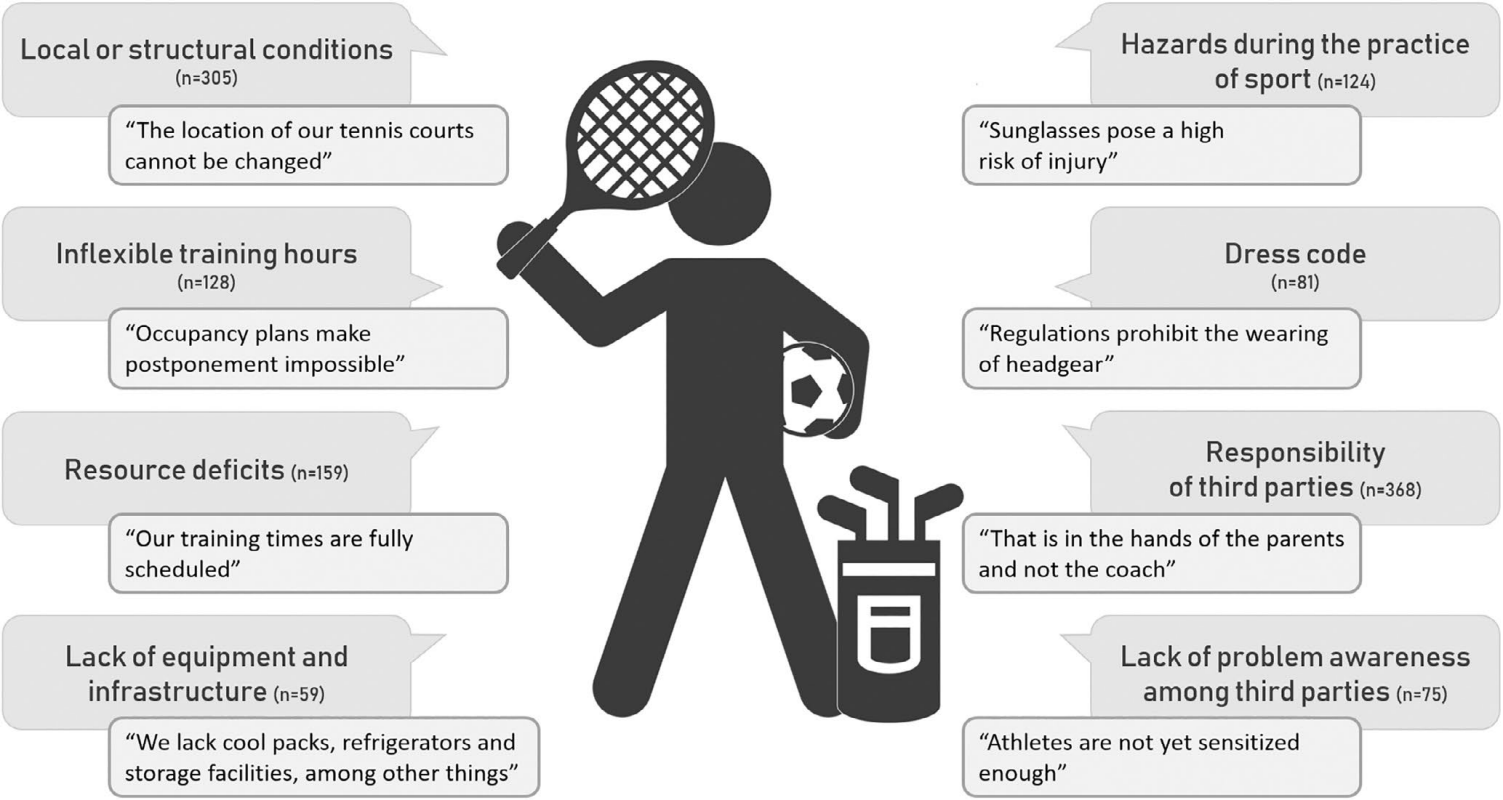
Reasons why UV prevention measures are impossible or difficult to implement. Legend: *n* = 1200. Speech bubble = category. Box = anchor example.

The open question as to why prevention measures are difficult or impossible to implement in the specific setting was answered by 772 coaches. The often very detailed reports contained 1299 individual arguments. The coaches most often denied responsibility and tended to see others, especially the athletes or their parents, as responsible. Further obstacles were the local and structural conditions, which were not easy to change. Fixed training times, dangers or obstacles due to protective measures and lack of staff, time and money were also frequently mentioned (Figure 3).

## 4 Discussion

Given that exposure times often last for hours, outdoor sporting environments are high risk exposure sites of UV radiation [4]. In this context, coaches are the most important key persons for the prevention of acute and chronic skin damage in athletes [3, 4, 6, 8].

There has long been an international consensus on suitable measures to protect against UV exposure [2, 4, 5, 16]. If the required measures (use of water‐resistant sunscreen with a sufficient sun protection factor, regular re‐application, wearing a hat, clothing that covers the shoulders, etc.) are operationalised with a score, a mean value of 53 out of a maximum of 100 points reveals clear deficits in German outdoor sports. There is untapped potential for prevention, particularly in the proper use of sunscreen, sunglasses, and shade, avoidance of midday sun and information about the current UV index. What makes our study unique is the identification of sport‐, coach‐, and club‐specific differences and opportunities for improvement. The sports of football, tennis, and swimming, as well as settings with inexperienced unlicensed coaches, in smaller clubs with smaller training groups and in children's and youth training, performed significantly worse in terms of protection from solar UV radiation. Self‐reported barriers to the implementation of recommended prevention measures range from local, structural, and temporal conditions, safety aspects and lack of resources (no staff, no time, no money) to shirking of responsibility.

This means that of all things, the two most popular outdoor sports in Germany, namely football and tennis, have the most striking prevention deficits. With 7.4 million members, the German Football Association [Deutscher Fußballbund] (DFB) is by far the largest sports association in Germany. The German Tennis Association [Deutscher Tennisbund] (DTB) follows in second place with 1.5 million members [10]. In both sports, league matches and tournaments often take place at midday in the summer under the blazing sun and without any shade on the pitch. The number of 2.6 million children and adolescents participating in these two sports illustrates the great public health relevance of this field of prevention: 18% of all German children and adolescents are active in these sports. Children and adolescents are particularly susceptible to UV radiation due to their sensitive skin structure [6, 17, 18]. According to Gomez Garcia et al. childrens' vulnerable epidermal stem cells are located closer to the skin surface and only migrate into deeper skin layers with the development of the terminal hairs [19, 20]. Therefore, sunburns in childhood increase the risk of later melanoma genesis in particular.

### 4.1 Limitations and Strengths of the Study

The weaknesses of this study include the non‐consideration of sport‐specific sun protection measures and latest product standards for sunscreens, the lack of comparison with the overall population, the possibility of typical survey biases, misinterpretation and limited validity: As a validated and standardized questionnaire based on the national medical guideline was used, it was not possible to take into account sport‐specific features of UV protection. So it makes sense for example, to apply sunscreen more frequently than every 2 h in sport, due to the sweat production of the skin and possibly also by drying off the sweat with towels. In addition, modern sunscreens are now able to provide complete UV protection much sooner than after 20 min. In addition, the score values could not be compared with other population groups in Germany due to a lack of data for the overall population. Furthermore, a selection effect cannot be ruled out, as it is possible that coaches who considered the topic relevant and were therefore supposedly more likely to pay attention to UV protection were more willing to participate. There is also the possibility of a recall bias and socially desirable response tendencies (so‐called social desirability bias) [16]. These types of bias typical of survey studies could mean that prevention deficits might de facto be even greater in the basic population [4, 16]. A further limitation results from the fact that some of the outdoor sports considered here are also practised indoors, for example in tennis, climbing or riding arenas or in covered velodromes. We did explicitly ask respondents to answer the questions for “typical outdoor training” on “a cloudless (summer) day.” However, misinterpretations cannot be ruled out. Finally, the external validity of our findings should be tested by examining any differences between reported and actual behaviour as well as the transferability of our results to other regions of the world—and thus other geographical latitudes—in future studies [4]. The strengths of our study include the large sample size, its representativeness and the ability to differentiate between the most relevant sports and other determinants in this context.

### 4.2 Classification Within the Research Context

Our findings on prevention are consistent with existing research on sunscreen use in sport, for example, that coaches do not emphasise preventive measures enough and athletes do not apply enough sunscreen [5, 6]. This is also supported by a recent representative study from the USA, which found that recreational athletes had a 5% higher incidence of multiple sunburns over a year than non‐athletes [17]. A recent representative study of German outdoor athletes showed that the incidence of erythema was higher in team sports such as football than in individual sports (21%, *p* = 0.024) [21].

The barriers to adequate prevention identified here (such as lack of awareness of the problem and shifting of responsibility) also show parallels with previous research: studies from the USA and Germany, for example, suggest a certain lack of concern and motivation for UV‐specific protective measures on the part of some coaches [3]. In group discussions on relevant prevention programmes for underage outdoor athletes in Germany, some coaches expressed the view that sun protection is more the individual responsibility of the young athletes [3]. In a study from the USA, three out of four athletes surveyed stated that they were never or rarely asked about sun protection by their coaches [6]. Such an attitude is controversial, as coaches are role models and authority figures who, among other things, decide on starting places and nominations [4, 5]. Furthermore, Marshall points out that such an attitude on the part of the coach is particularly fatal for young athletes, as they often do not have the courage to interrupt their training—for example during structured exercise drills—to take shadow breaks, for example [22].

In a current systematic scoping review, it is criticised that despite the explosive nature of the topic, hardly any studies are available on the subject of “sun protection in sport” [4]. The situation is quite different regarding another area of prevention in outdoor sports: The topic of external heat illnesses. In the USA and Australia, there are long‐established position statements, guidelines, and recommendations for the prevention and treatment of external heat illness, which are also extensively monitored and evaluated [18, 23].

The recently published “SC^3^ Pyramid‐Model” suggests combining the prevention of heat‐related illnesses and UV damage in sport with the implementation of structural measures. The preventive measures proposed in the model include technical and structural measures (e.g., installation of awnings over training areas and substitution benches, provision of sunshades by coaches during training and match breaks) [24, 25, 26], organisational measures (e.g., avoidance of midday sun, exclusion of athletes without adequate sun protection, rule changes, clothing policy, additional breaks in the shade, redesign of endurance events as night runs, etc.) [3, 4, 24, 25, 26, 27], and personalised measures (e.g., purchase of certified jerseys and sportswear with UPF ratings, stickers in changing rooms reminding athletes to use sunscreen, provision of sunscreen dispensers) [3, 4, 26].

In the course of climate change, solar UV risks will continue to increase [28]. This applies in particular to the risk setting of outdoor sports. If we want to continue to enjoy these sports successfully and safely in the future, existing prevention deficits should be addressed urgently and comprehensively—in stadiums, on sports fields, in the mountains and on the water.

## Author Contributions

Conceptualization: S.S. and S.L. Methodology: S.S., S.L. and C.E. Resources: S.S., S.L. and C.E. Writing – original draft preparation: S.S. and S.L. Writing – review and editing: S.S., S.L. Visualization: S.S. and S.L. Translation: S.L. Supervision: S.S. Project administration: S.S., S.L. All authors have read and agreed to the published version of the manuscript.

## Conflicts of Interest

The authors declare no conflicts of interest.

## Data Availability

The original data that support the findings of this study are available in German on request from the corresponding author (S.S).
